# Obstructive Sleep Apnea Impacts Cardiac Function in Dilated Cardiomyopathy Patients Through Circulating Exosomes

**DOI:** 10.3389/fcvm.2022.699764

**Published:** 2022-03-07

**Authors:** Hui Gong, Xing Lyu, Lini Dong, Shengyu Tan, Shizhen Li, Jieting Peng, Yang Liu, Xiangyu Zhang

**Affiliations:** ^1^Department of Geriatrics, The Second Xiangya Hospital, Central South University, Changsha, China; ^2^Laboratory of Clinical Medicine, The Second Xiangya Hospital, Central South University, Changsha, China

**Keywords:** obstructive sleep apnea, heart failure, exosomes, autophagy, Dilated Cardiomyopathy

## Abstract

**Background:**

Obstructive sleep apnea (OSA) is common and independently associated with heart failure. This study aimed to investigate the impact of OSA on heart function in patients with dilated cardiomyopathy (DCM) as well as the possible mechanism related to exosomes regulated autophagy.

**Methods and Results:**

A total of 126 patients with DCM who underwent sleep evaluations were analyzed retrospectively. Cardiomyocytes were treated with exosomes isolated from untreated OSA patients and healthy controls. Fibrotic and hypertrophic markers were evaluated, and Akt/mTOR pathway-mediated autophagy was investigated. DCM patients with severe OSA had larger right ventricular end-diastolic diameter (RVEDd) and right atrial diameter (RAD) and increased N-terminal pro-B-type natriuretic peptide (NT-proBNP) levels than DCM patients without OSA. Moreover, NT-proBNP and diabetes mellitus were independently correlated with the apnea-hypopnea index in multiple linear regression analysis. Treatment with OSA-derived exosomes significantly increased Col1A1, ANP, and BNP protein expression and decreased the expression of the autophagy markers LC3B II/I and beclin1. Rapamycin treatment significantly increased the decreased autophagy markers and attenuated the increased expression of Col1A1, ANP and BNP induced by OSA-derived exosomes.

**Conclusion:**

The severity of OSA is significantly associated with cardiac injury and remodeling. The underlying mechanism may be related to changed autophagy levels, which are regulated by circulating exosomes *via* the Akt/mTOR signaling pathway. This study may provide a new clue for the treatment of heart failure with OSA.

## Introduction

Dilated cardiomyopathy (DCM) is the leading cause of heart failure (HF), which accounts for 60% of HF (1, 2). Without treatment, the 1-year survival rate is 70–75%, and the 5-year survival rate is as low as 50% (3, 4). Many factors contribute to the development and progression of HF; obstructive sleep apnea (OSA) is among these factors.

OSA is a sleeping disorder caused by repeated obstruction of the upper airway during nighttime sleep, resulting in episodes of interrupted respiration, fragmented sleep and intermittent hypoxia (IH) (5). Accumulating evidence has demonstrated that OSA is closely associated with an increased risk of cardiovascular disease, including HF. The prevalence of OSA is substantially increased in HF patients (6), and OSA is also a poor prognostic indicator for HF patients (7).

The concurrence of OSA and DCM is not uncommon in clinical practice (8–10), but this phenomenon has not received sufficient attention. Only a few studies have reported that OSA may contribute to impaired left ventricular function in patients with DCM, and nasal continuous positive airway pressure (CPAP) can significantly improve the symptoms and left ventricular ejection fraction (LVEF). The relationship of cardiac function in DCM patients with OSA severity was not indicated (10, 11). In this context, we retrospectively analyzed the clinical characteristics of DCM patients with and without OSA to understand whether the different degrees of OSA are associated with changes in heart structure and function.

Although CPAP is generally employed to treat OSA, the prognosis of HF with OSA remains poor. This observation suggests that in addition to mechanical obstruction, other mechanisms, such as endocrine, paracrine and autocrine changes induced by OSA, may also exert a role in the pathophysiology of HF (12, 13). Circulating microparticles (MPs) can induce endothelial dysfunction by reducing endothelial-derived nitric oxide production (14), and discontinuation of CPAP therapy leads to a significant increase in endothelium-derived MP levels in OSA patients (15). These studies provide evidence that circulating extracellular vesicles may be important messengers linking OSA to cardiovascular diseases.

Exosomes are types of extracellular vesicles that range between 30 and 100 nm in size. Exosomes are present in almost all biological fluids, including blood, urine, saliva, cerebrospinal fluid, and breast milk (16, 17). Exosomes carry numerous biologically active substances, such as cytokines, proteins, lipids, mRNAs, microRNAs and many other non-coding RNAs. Exosomes play significant roles in mediating intercellular communication *via* these cargoes (18, 19). Some exosomes can be released into the blood circulation and are known as circulating exosome (20). These exosomes reach tissues through blood circulation and directly interact with target cells and exert biological functions (21). Studies have shown that exosomes play a role in cardiovascular diseases, including HF (22). It has been demonstrated that IH exposure induces exosomes release into the circulation and promote increased permeability and dysfunction of endothelial cells in healthy young adults (23); circulating exosomes in untreated OSA produce a significant increase in endothelial cell senescence, which is an important contributor of end-organ dysfunction (24). Thus, we hypothesize that IH-induced changes in circulating exosomes would also disrupt the structure and function of cardiomyocytes, which represents the pathological basis of HF. Therefore, we isolated plasma exosomes from untreated OSA patients and investigated their effects on markers of cardiomyocyte damage *in vitro*.

## Methods

### Human Subjects

One hundred and twenty-six DCM patients with polysomnography (PSG) data hospitalized in the Second Xiangya Hospital from 2015 to 2020 were retrospectively analyzed. Among these patients, PSG was performed due to the clinical suspicion of OSA based on the presence of obesity and a history of loud snoring and daytime somnolence. The diagnostic criteria of DCM were consistent with the 2013 American Heart Association/American College of Cardiology guidelines (25). Exclusion criteria included the following: (1) ischemic cardiomyopathy; (2) valvular heart disease; (3) obstructive lung disease; (4) renal function impairment; and (5) history of malignancy. OSA was defined as the number of apnea-hypopnea index (AHI) ≥ 5 events per hour of sleep. The severity of OSA was evaluated based on the AHI. According to the AHI results, the DCM patients were divided into three groups: no OSA group (AHI <5.0 events per hour), mild to moderate OSA group (AHI: 5.0–29.9 events per hour), and severe OSA group (AHI ≥ 30.0 events per hour). The demographics, complications and electrocardiograms of the patients were retrospectively reviewed and recorded. This study was approved by the institutional ethics committee of the Second Xiangya Hospital of Central South University.

In the part of cell experiments, Exosomes were isolated from peripheral venous blood of 10 OSA patients and 10 healthy controls. Individuals aged 18–60 years who underwent PSG in the physical examination center were enrolled. Exclusion criteria included cardiovascular heart diseases, hepatic or renal dysfunction, acute infection, autoimmune diseases, cancer, metabolic syndrome, etc. All subjects were informed of the purpose, risks, and benefits of the study and signed informed consent forms.

### Polysomnography (PSG)

An overnight PSG was performed in all subjects using standard technique (SOMNO lab2, Weinmann, Germany). Prior to sleep study, body weight, body height, body mass index (BMI), and neck size were measured. The PSG device includes oximetry, thermistor measurements of airflow, electroencephalography, electromyography, electrooculography, and measurements of costal and abdominal movements when breathing. The respiratory events were identified by experienced technicians according to the standard criteria. The hourly number of episodes of apnea and hypopnea was calculated as AHI. All data were recorded on a computerized sleep scoring system.

### Echocardiography

Echocardiographic images were collected in the parasternal long and short axis, the apical long axis, and the apical four chamber view. The measurements of left ventricular end-diastolic diameter (LVEDd), left atrial diameter (LAD), right ventricular end-diastolic diameter (RVEDd), right atrial diameter (RAD), and LVEF were determined following the American Society of Echocardiography recommendations.

### Circulating Plasma Exosome Preparation and Cardiomyocyte Culture

Exosomes were isolated from peripheral venous blood of OSA patients (OSA-Exos) and healthy controls (Ctrl-Exos) using the Exoquick TC exosome isolation kit (Echo9101A) according to the manufacturer's standard protocol. PSG were performed in all subjects. The number of OSA-Exos in each sample was determined using a BCA protein assay kit (Beyotime) as described by the manufacturer's guidelines. The isolated exosomes were characterized by Western blot analysis of the exosome markers CD9, CD81, TSG101, and Calnexin. A NanoSight instrument (Zeta VIEW, Germany) and Zeta View 8.04.02 software was used for nanoparticle tracking analysis (NTA). Exosome morphology was observed directly under a transmission electron microscope (FEI Tecnai G2 Spirit).

The rat cardiomyocyte cell line H_9_C_2_ was purchased from the Cell Bank of China Science Academy (Shanghai, China). Cells were maintained in Dulbecco's modified Eagle medium (DMEM) with 10% fetal bovine serum (FBS) and 1% penicillin/streptomycin (15140-122, Gibco, China) in a humidified incubator at 37°C and 5% CO_2_ atmosphere. H_9_C_2_ cells were treated with OSA-Exos or Ctrl-Exos for 24 h.

### Western Blot Analysis

Total protein was extracted from H_9_C_2_ cells using a total protein extraction kit (Beyotime). A BCA protein assay kit was used to measure the protein concentration. Each sample (10 μg) was subject to 10 or 12% sodium dodecyl sulfate polyacrylamide gel electrophoresis and then transferred to polyvinylidene difluoride membranes (Millipore, Bedford, MA). Membranes were blocked with 5% milk for 1 h at room temperature and incubated with the following primary antibodies: CD9, CD81, TSG101, Calnexin (Abcam, Cambridge, UK; #ab275018, dilution 1:1000), Collagen 1A1 (Col1A1) (CST; #72026, dilution 1:1000), atrial natriuretic peptide (ANP) (Abcam, #ab225844, dilution 1:1000), brain natriuretic peptide (BNP) (Abcam, #ab92500, dilution 1:1000), LC3B (Abcam, Cambridge, UK; #ab48394, dilution 1:1000), beclin1 (Abcam; ab210498, dilution 1:1000), Akt (CST; #4691, dilution 1:1000), p-Akt (CST; #4060, dilution 1:1000), mTOR (CST; #2983, dilution 1:1000), p-mTOR (CST; #5536, dilution 1:1000) and GAPDH (Abcam, Cambridge, UK; #ab9485, dilution 1:5000) at 4°C overnight. After 3 washes with TBST, the membranes were incubated with secondary antibodies for 1 h at room temperature. Finally, the membranes were visualized using a chemiluminescence detection system.

### Immunofluorescence Staining

H_9_C_2_ cells were plated in 35 mm dishes, washed with PBS and then fixed with 4% paraformaldehyde for 20 mins at room temperature. After being washed thrice with PBS, the H_9_C_2_ cells were blocked in 5% goat serum for 1 h. The cells were subsequently incubated with primary antibodies specific for Col1A1 (1:400, Abcam) overnight at 4°C. On the next day, the H_9_C_2_ cells were washed with PBS and incubated with fluorescence-conjugated secondary antibodies (Beyotime, China) followed by DAPI. Images were captured under a fluorescence microscope.

### Statistical Analysis

SPSS 22.0 statistical software was used for statistical analysis. Quantitative data are expressed as the mean value ± standard deviation (SD). Continuous variables with non-normal distributions are presented as median values (interquartile range), and qualitative variables are presented as frequencies. Normally distributed data were compared using Student's *t*-test between two groups, and one-way analysis of variance (ANOVA) was used to compare the differences among multiple groups. Spearman correlation analysis was performed to determine the correlation between two factors. Variables with *p* < 0.05 on univariate analysis were entered into multivariate linear regression models to determine the factors associated with HF. All reported probability values were two-tailed, and a *p*-value <0.05 was considered statistically significant.

## Results

### Demographic and Clinical Characteristics of the Patients

The baseline demographic and clinical characteristics of the study population are shown in Table 1. A total of 126 DCM patients were enrolled. According to the PSG results, 15 DCM patients had no OSA, 50 had mild to moderate OSA, and 61 had severe OSA. The mean AHI values were 3.1 (1.9, 3.6) vs. 15.1 (10.0, 24.4) vs. 41.5 (33.7, 48.9) (*p* < 0.05), respectively. The prevalence of OSA was significantly greater in male subjects than in female subjects, and DCM patients with mild to moderate were older than those without OSA in this study. Compared to patients without OSA and mild to moderate OSA patients, body mass index (BMI) and N-terminal pro-B-type natriuretic peptide (NT-proBNP) levels were significantly increased in severe OSA patients. DCM patients with severe OSA had the greatest prevalence of hypertension and diabetes mellitus. No significant differences in fasting plasma glucose (FPG), systolic blood pressure (SBP), diastolic blood pressure (DBP), or the New York Heart Association functional classification (NYHA class) were observed among the three groups. LVEDd, LAD and LVEF were not significantly different among the three groups. RVEDd and RAD were similar in the non-OSA group and the mild to moderate OSA group, whereas these parameters were significantly greater in severe OSA patients compared with two other groups. Regarding guideline guided medical therapy, including angiotensin converting enzyme inhibitors, angiotensin receptor blockers, angiotensin receptor-neprilysin inhibitors, beta receptor blockers, spironolactone, Furosemide/Torasemide, and digoxin, there was no significant difference among the three groups.

**Table 1:** Baseline characteristic, echocardiographic data, and medical therapy of all patients.

**Variables**	**Non-OSA group** **(*n* = 15)**	**Mild to moderate OSA group** **(*n* = 50)**	**Severe OSA group** **(*n* = 61)**	***p* value**
**Demographics**				
Age, years	42 (31, 57)	52.5 (42.5, 57.3)^*^	44.0 (31.5, 52.0)	0.015
Sex (Male, %)	14 (93.3)	46 (92)	58 (95.1)	0.812
BMI (kg/m^2^)	27.1 (25.1, 29.3)	27.6 (25.5, 31.6)	29.9 (27.6, 35.6) ^#^	0.045
**Blood pressure**				
SBP (mmHg)	122.2 ± 16.6	125.1 ± 17.6	127.4 ± 19.4	0.560
DBP (mmHg)	82.7 ± 12.0	83.6 ± 14.7	88.4 ± 15.4	0.162
**Blood test results**				
TG (mmol/L)	1.34 (0.95, 2.44)	1.58 (1.23, 2.78) ^*^	1.40 (0.86, 1.98)	0.122
TC (mmol/L)	3.96 (3.54, 4.61)	4.02 (3.20, 4.54)	3.55 (3.13, 4.40)	0.500
HDL-C (mmol/L)	0.96 (0.89, 1.10)	0.94 (0.72, 1.11)	0.85 (0.74, 0.97)	0.205
LDL-C (mmol/L)	2.54 (1.96, 2.93)	2.50 (1.91, 3.12)	2.41 (1.88, 2.96)	0.789
FPG (mmol/L)	5.4 (5.1, 6.0)	5.50 (5.08, 6.60)	5.70 (5.20, 7.30)	0.432
NT-proBNP (pg/ml)	804 (406,1110)	801 (591,1347)	2889 (2227,5126) ^#^	0.000
**NYHA class**				
NYHA I (%)	0 (0)	1 (2)	0 (0)	0.267
NYHA II (%)	4 (26.7)	7 (14)	8 (13.1)	0.892
NYHA III (%)	8 (53.3)	22 (44)	33 (54.1)	0.290
NYHA IV (%)	3 (20)	19 (38)	20 (32.8)	0.567
**Medical history and cardiovascular risk factors**				
Hypertension (%)	4 (26.7)	21 (42)^*^	36 (59.0)^#^	0.034
Diabetes mellitus (%)	2 (13.3)	7 (14)	21 (34.4)^#^	0.121
Current smoking (%)	7 (46.7)	29 (58)	34 (55.7)	0.567
Current drinking (%)	2 (13.3)	13 (26)	19 (31.1)	0.188
**Echocardiographic data**				
LVEDd (mm)	65.7 ± 6.8	66.5 ± 8.1	68.6 ± 7.2	0.315
LAD (mm)	45.4 ± 6.6	47.1 ± 8.4	48.8 ± 6.5	0.200
RAD (mm)	35.0 (33.0, 38.0)	35.5 (33.0, 42.0)	40.0 (35.5, 45.5)^#^	0.015
RVEDd (mm)	35.0 (30.0, 39.0)	34.0 (30.0, 42.3)	39.0 (35.0, 45.0)^#^	0.000
LVEF (%)	35.4 ± 8.8	34.3 ± 8.7	32.5 ± 8.2	0.366
**Medical therapy**				
Beta-blockers (%)	15 (100)	45 (88.2)	56 (91.8)	0.419
ACEIs/ARBs (%)	9 (60)	38 (76)	43 (70.5)	0.328
ARNI (%)	6 (40)	13 (26)	15 (24.6)	0.363
Spironolactone (%)	15 (100)	40 (80)	55 (90.2)	0.057
Furosemide/Torasemide (%)	10 (66.7)	35 (70)	51 (83.6)	0.163
Digoxin (%)	4 (26.7)	8 (16)	16 (26.2)	0.341

*Data are expressed as means ± standard deviation for normally distributed data, median (interquartile range) for non-normally distributed data, or number (%) for categorical variables. OSA, obstructive sleep apnea; BMI, body mass index; SBP, systolic blood pressure; DBP, diastolic blood pressure; TG, triglyceride; TC, total cholesterol; HDL-C, high-density lipoprotein cholesterol; LDL-C, low-density lipoprotein cholesterol; FPG, fasting plasma glucose; NT-pro BNP, N-terminal pro-brain natriuretic peptide; LVEDd, left ventricular end-diastolic diameter; LAD, left atrial diameter; RAD, right atrial diameter; RVEDd, right ventricular end-diastolic diameter; LVEF, left ventricular ejection fraction. ACEIs, angiotensin-converting enzyme inhibitors; ARBs, angiotensin II receptor blockers; ARNI, angiotensin receptor-neprilysin inhibitor*.

* *p < 0.05, compared to non-OSA group*;

# *p < 0.05, compared to non-OSA or mild to moderate OSA group*.

### Correlations of Patients' Clinical Parameters With the AHI

Spearman correlation analysis was performed to determine factors that correlated with the AHI. The results revealed that BMI (*r* = 0.243, *p* = 0.009), NT-proBNP (*r* = 0.512, *p* = 0.001), RVEDd (*r* = 0.292, *p* = 0.001), RAD (*r* = 0.188, *p* = 0.039), hypertension (*r* = 0.216, *p* = 0.017) and diabetes mellitus (*r* = 0.245, *p* = 0.007) were positively correlated and age (*r* = −0.212, *p* = 0.02) and HDL-C (*r* = −0.187, *p* = 0.04) were negatively correlated with the AHI. Sex, TG, TC, LDL-C, LVEDd, LAD, and LVEF had no correlation with the AHI. Multiple linear regression analysis was performed to further evaluate the correlation of age, BMI, HDL-C, NT-proBNP, RVEDd, RAD, hypertension and diabetes mellitus with the AHI. The results revealed that NT-proBNP and diabetes mellitus remained independently associated with the AHI (Table 2).

**Table 2:** Spearman correlation and multiple linear regression analysis of the factors associated with AHI.

**Variable**	**Spearman correlation**		**Multivariate Adjusted**	
	**analysis**		**R**^**2**^ **= 0.158**	
	***r***	***p***	**β**	***p***
Age	−0.212	0.020		
Sex	−0.102	0.266		
BMI	0.243	0.009		
TG	−0.135	0.140		
TC	−0.130	0.158		
HDL-C	−0.187	0.040		
LDL-C	−0.110	0.231		
NT-proBNP	0.512	0.001	0.002	0.039
LVEDd	0.157	0.086		
LAD	0.134	0.143		
RVEDd	0.292	0.001		
RAD	0.188	0.039		
LVEF	−0.123	0.177		
Hypertension	0.216	0.017		
Diabetes mellitus	0.245	0.007	11.150	0.002

*AHI, apnea-hypopnea index; BMI, body mass index; TG, triglyceride; TC, total cholesterol; HDL-C, high-density lipoprotein cholesterol; LDL-C, low-density lipoprotein cholesterol; LVEDd, left ventricular end-diastolic diameter; LAD, left atrial diameter; RVEDd, right ventricular end-diastolic diameter; RAD, right atrial diameter; LVEF, left ventricular ejection fraction; NT-proBNP, N-terminal pro-brain natriuretic peptide*.

### Exosomes Characterization

Exosomes were extracted from untreated OSA patients and healthy controls. The characteristics of the subjects are shown in Supplementary Table 1. Age, BMI and AHI were higher in OSA patients than in controls. Thus, a multivariate stepwise regression analysis was performed to correct the differences between the two groups. The results showed that only AHI (standardized β = 0.814, 95% confidence interval [CI]: (0.012,0.027), *p* = 0.000) was significantly associated with the presence of OSA (Supplementary Table 2). This analysis showed that age and BMI had no effect on the results in this study.

**Figure 1:**
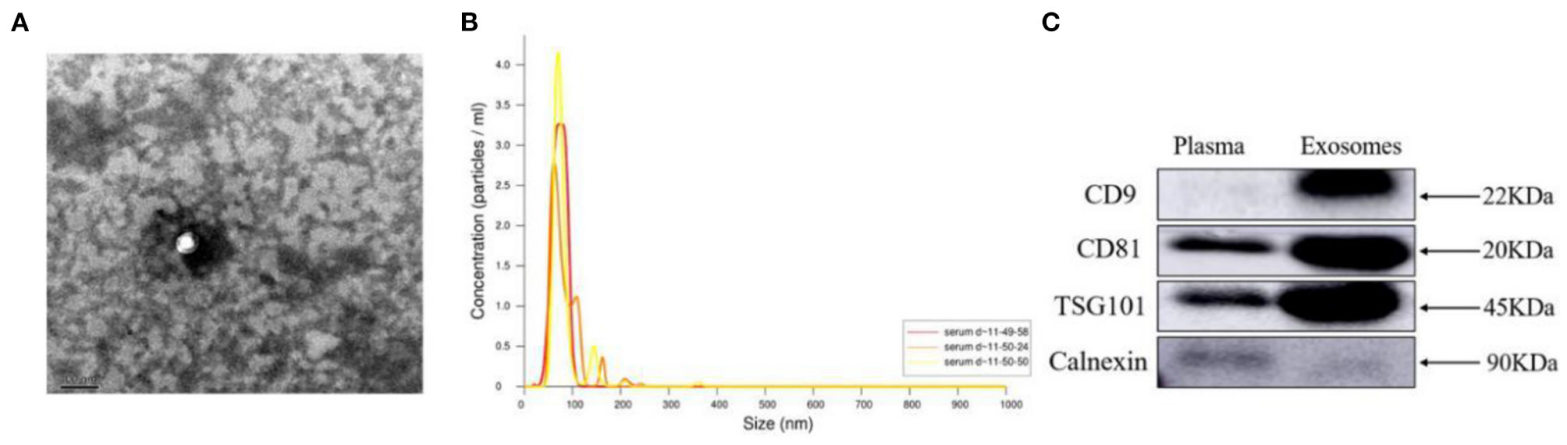
Identification of isolated plasma exosomes. **(A)** Image of isolated exosomes under a transmission electron microscope. Scale bar = 100 nm. **(B)** The mean diameter was ~79.2 nm, and the particle concentration was 1.24 × 10^9^ particles/ml as determined by NanoSight analysis. Horizontal axis, particle size (nm); vertical axis, particle concentration (particles/ml). **(C)** Protein levels of exosomal positive markers CD9, CD81 and TSG101 were significantly increased and negative marker Calnexin was significantly reduced in the exosomes group compared with the plasma group.

Exosome morphology was observed by transmission electron microscopy. The image showed that the vesicles had a typical cup shape with a diameter of ~100 nm (Figure 1A). NanoSight analysis showed that the mean diameter of exosomes was 79.2 nm, and the concentration was 1.24 × 10^9^ particles/ml (Figure 1B). Exosome surface markers were detected by Western blot. The results showed that the exosomal positive markers CD9, CD81 and TSG101 were significantly higher and the negative marker Calnexin was significantly lower in the exosomes group than in the plasma group (Figure 1C). These results suggested that exosomes were purified successfully from plasma in our experiments.

### Effects of OSA-Derived Exosomes on Fibrotic and Hypertrophic Markers in Cardiomyocytes

**Figure 2:**
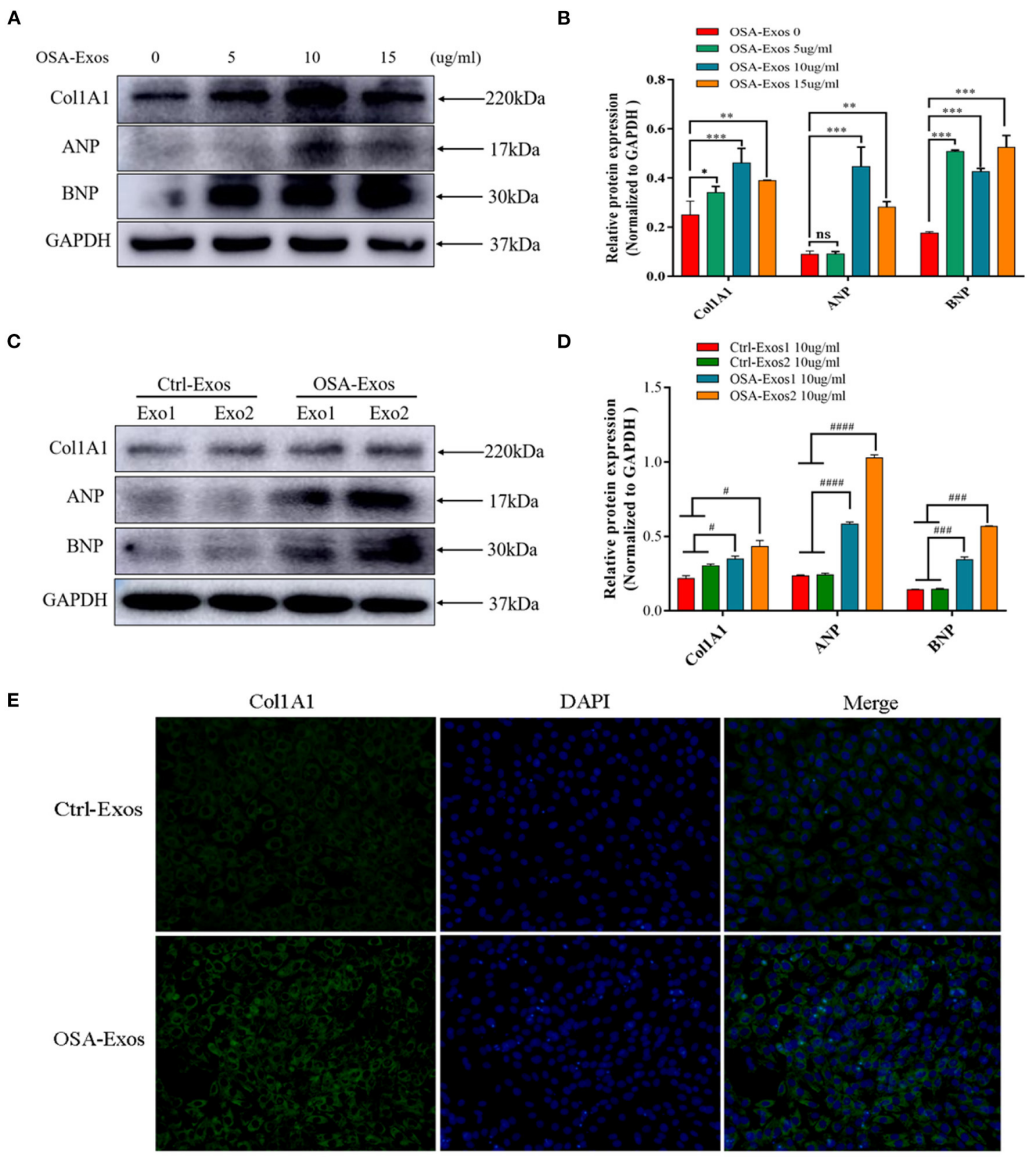
Exosomes from OSA patients (OSA-Exos) increased the expression of Col1A1, ANP and BNP in H_9_C_2_ cells. **(A)** Col1A1 and BNP protein expression was significantly increased in H_9_C_2_ cells after treatment with 5, 10, and 15 μg/ml exosomes from one OSA patient compared with the control (0 μg/ml); ANP protein expression was significantly increased in H_9_C_2_ cells after treatment with 10 and 15 μg/ml exosomes compared with the control (0 μg/ml). **(B)** Densitometric analyses of the Western blot presented in A. **(C)** Col1A1, ANP and BNP protein expression was significantly increased in H_9_C_2_ cells treated with 10 μg/ml OSA-Exos compared with exosomes from healthy controls (Ctrl-Exos) as assessed by Western blot. **(D)** Densitometric analyses of the Western blot presented in C. GAPDH served as the internal control. **(E)** H_9_C_2_ cells were treated with 10 μg/ml OSA-Exos and Ctrl-Exos. Col1A1 (green) expression was detected by immunofluorescence staining. The bars indicate the mean ± SD of three separate experiments. *p < 0.05, **p < 0.01, ***p < 0.001 vs. OSA-Exos 0 group. ^#^*p* < 0.05, ^###^*p* < 0.001, ^####^*p* < 0.0001 vs. Ctrl-Exos groups. ns, no significant.

The effects of exosomes derived from the plasma of OSA patients and healthy controls on cardiomyocytes were examined, and fibrotic and hypertrophic markers were evaluated. First, exosomes from the same OSA patient (OSA-Exos) at the protein concentrations of 0 (control), 5, 10, and 15 μg/ml were used to treat H_9_C_2_ cells for 24 h. The results showed that 5, 10, and 15 μg/ml OSA-Exos treatments significantly increased the expression of the fibrotic marker Col1A1 and hypertrophic markers ANP and BNP compared to the 0 μg/ml (control), and the highest level was observed with 10 μg/ml exosomes treatment (Figures 2A,B). Thus, 10 μg/ml exosomes in the culture media were used in the subsequent experiments. To further explore the pro-fibrotic and pro-hypertrophic effects of OSA-Exos on cardiomyocytes, H_9_C_2_ cells were treated with exosomes from different OSA patients and healthy controls (Ctrl-Exos) at a concentration of 10 μg/ml for 24 h. Col1A1, ANP and BNP expression was significantly increased in the OSA-Exos-treated group compared with the Ctrl-Exos group (Figures 2C,D). Additionally, robust accumulation of Col1A1-positive cells was observed by immunofluorescence staining in the OSA-Exos group (Figure 2E). These results indicated that OSA-Exos may contribute to cardiomyocyte damage.

### OSA-Derived Exosomes Inhibited Akt/MTOR-Mediated Autophagy in Cardiomyocytes

**Figure 3:**
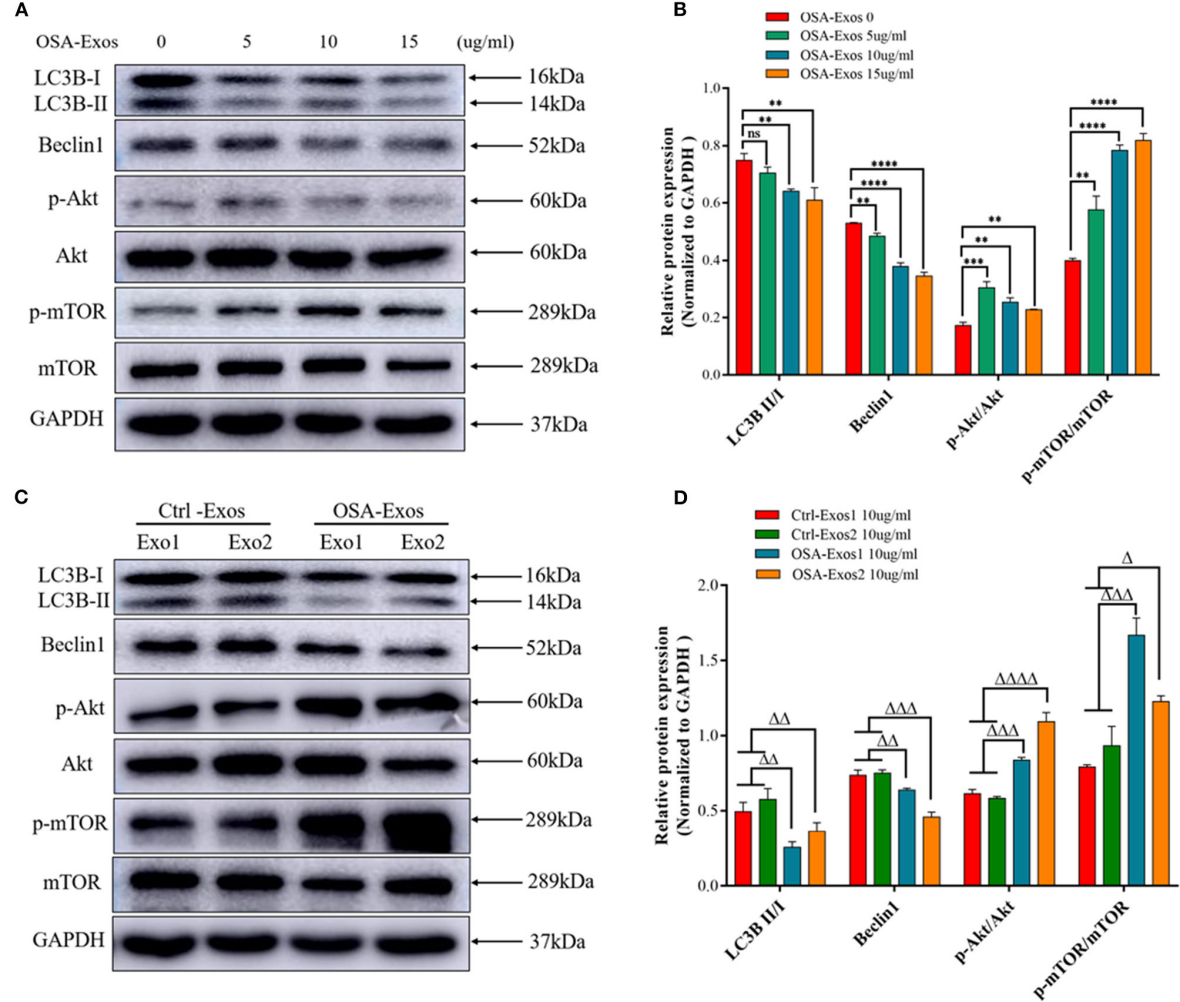
OSA-Exos inhibited autophagy *via* the Akt/mTOR signaling pathway in H_9_C_2_ cells. **(A)** LC3B II/I expression decreased in H_9_C_2_ cells treated with 10 and 15 μg/ml exosomes from one OSA patient compared with the 0 (control) and 5 μg/ml. Beclin1 protein expression decreased, p-Akt/Akt and p-mTOR/mTOR expression increased in H_9_C_2_ cells treated with 5, 10 and 15 μg/ml exosomes compared with the control (0 μg/ml). **(B)** Densitometric analyses of the Western blot presented in A. **(C)** LC3B II/I and beclin1 protein expression decreased, p-Akt/Akt and p-mTOR/mTOR expression increased in H_9_C_2_ cells treated with 10 μg/ml OSA-Exos compared with the control as determined by Western blot analysis. **(D)** Densitometric analyses of the Western blot presented in C. GAPDH served as the internal control. The bars indicate the mean ± SD of three separate experiments. **p < 0.01, ***p < 0.001, ****p < 0.0001 vs. OSA-Exos 0 group. ^Δ^*p* < 0.05, ^ΔΔ^*p* < 0.01, ^ΔΔΔ^*p* < 0.001, ^ΔΔ*ΔΔ*^*p* < 0.0001 vs. Ctrl-Exos groups. ns, no significant.

Autophagy has been demonstrated to play a role in controlling the hypertrophic response of cardiomyocytes in previous studies, so we first detected the autophagy protein markers light chain 3B (LC3B) and beclin1 in this study. The results showed that LC3B II/I and beclin1 expression decreased after 5, 10, and 15 μg/ml OSA-Exos treatment (Figures 3A,B). When treated with exosomes from different OSA patients and healthy controls at a concentration of 10 μg/ml for 24 h, the expression of LC3B II/I and beclin1 was also significantly lower in the OSA-Exos group than in the Ctrl-Exos groups (Figures 3C,D). These results suggested that autophagy was inhibited by OSA-Exos and may be involved in the process of cardiomyocyte injury.

The Akt/mTOR pathway is a major negative regulator of autophagy (26). We then determined the expression of Akt and mTOR after treatment with OSA-Exos. As shown in Figures 3A,B, higher levels of p-Akt/Akt and p-mTOR/mTOR were observed after treatment with 5, 10, and 15 μg/ml OSA-Exos than in the control (0 μg/ml) from the same OSA patient. P-Akt/Akt and p-mTOR/mTOR were also increased in OSA-Exos from different OSA patients compared with healthy controls (Figures 3C,D). These results suggested that Akt/mTOR-mediated autophagy was inhibited by OSA-Exos and may participate in OSA-Exos induced cardiomyocyte injury.

### Akt/MTOR Pathway-Regulated Autophagy Was Involved in OSA-Exos Induced Cardiomyocyte Injury

**Figure 4:**
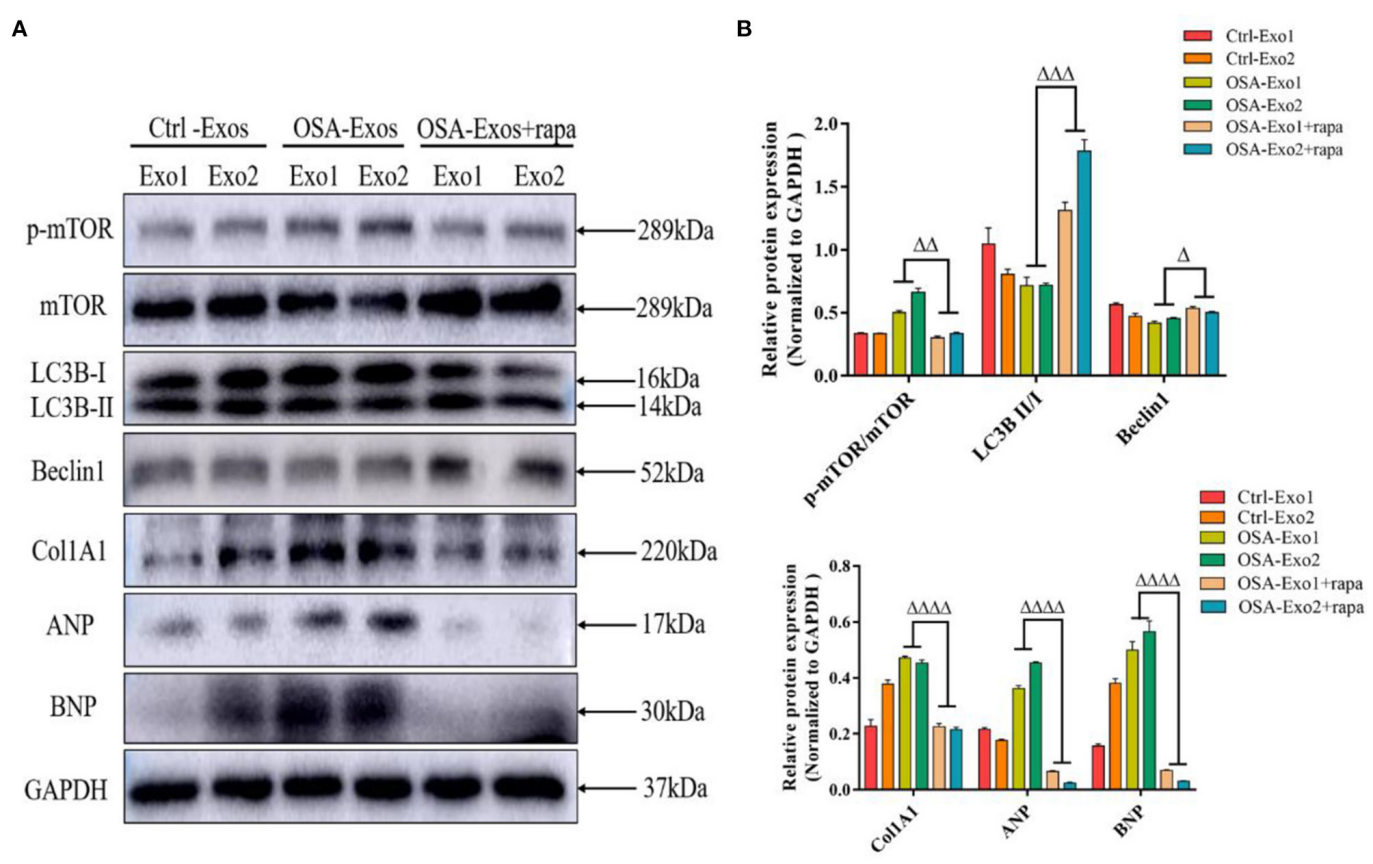
Akt/mTOR-mediated autophagy was involved in OSA-Exos induced cardiomyocyte damage. **(A)** Rapamycin treatment significantly decreased the increased p-mTOR/mTOR, Col1A1, ANP, and BNP protein expression and increased the decreased LC3B II/I and beclin1 expression induced by OSA-Exos treatment. **(B)** Densitometric analyses of the Western blot presented in A. GAPDH served as the internal control. The bars indicate the mean ± SD of three separate experiments. ^Δ^*p* < 0.05, ^ΔΔ^*p* < 0.01, ^ΔΔΔ^*p* < 0.001, ^ΔΔΔΔ^*p* < 0.0001 vs. OSA-Exos groups. rapa, rapamycin.

Rapamycin is an inhibitor of mTOR and an autophagy inducer. Rapamycin was used to further verify whether Akt/mTOR was involved in OSA-Exos induced cardiomyocyte injury. As shown in Figure 4, rapamycin treatment significantly decreased the increased p-mTOR/TOR expression and exacerbated OSA-derived exosome-induced inhibition of LC3B II/I and beclin1 expression. Furthermore, rapamycin significantly inhibited the expression of Col1A1, ANP and BNP protein induced by OSA-Exos treatment. These results demonstrated that OSA-Exos promoted cardiomyocyte injury could be alleviated by elevated autophagy levels through the Akt/mTOR signaling pathway.

## Discussion

In our study, we found that DCM patients with severe OSA had larger RVEDd and RAD and increased NT-proBNP levels compared with DCM patients without OSA. In contrast, LVEDd, LAD and LVEF had no significant differences among the three groups. Moreover, NT-proBNP and diabetes mellitus were independently correlated with the AHI in multiple linear regression analysis. These findings suggest that OSA may be a significant risk factor for HF. In addition, we provided evidence that circulating exosomes derived from untreated OSA patients increased myocardial fibrosis and hypertrophy by inhibiting autophagy through the Akt/mTOR signaling pathway. Thus, OSA might trigger or exacerbate HF *via* exosomes through autophagy-related pathways.

HF is the leading cause of hospitalization in elderly patients (13), and the mortality rate remains high despite great progress in treatment. Because PSG was performed based on the history of obesity, loud snoring and daytime somnolence, >90% of OSA patients were male in this study. This finding indicated that the prevalence of OSA in males is greater than that in females. In addition, the patients with OSA had a higher BMI and higher prevalence of diabetes mellitus than those without OSA. These results were consistent with previous studies showing that the prevalence and severity of OSA were significantly related to male sex, obesity, and diabetes mellitus (27, 28).

OSA is associated with cardiac functional and structural changes. During obstructive apnea, negative intrathoracic pressure was generated against an occluded upper airway, thereby increasing the left ventricle transmural pressure, an important determinant of left ventricular afterload (29). Consistent with this notion, Malone and colleagues illustrated that OSA contributes to impaired left ventricular function in DCM patients, and the reversal of OSA by nasal CPAP can improve LVEF (11). Our study found enlarged LVEDd and LAD in patients with DCM and OSA compared with DCM patients without OSA, although these variables were not statistically significant. We hypothesized that the discrepancy between our study and others arose from the smaller sample size. In contrast, we found that the RVEDd and RAD were significantly greater in severe OSA patients than in non-OSA patients and were associated with the AHI. This finding may be explained by the fact that during apnea, futile inspiratory efforts against the occluded pharynx cause a reduction in intrathoracic pressure and enhancement in venous return, inducing a leftward shift of the interventricular septum and right ventricle dilatation (30, 31). Repeated and periodic apneic episodes eventually lead to enlargement of the right ventricle and atrium. Early cineradiographic studies in humans and dogs also showed increased right heart size during the apneic episodes (32). This finding reflects that OSA was associated with right cardiac structural changes. In addition, considering that the cavity of the right ventricle has an irregular geometry with thin walls, the inflow tract and outflow tract are not in the same plane, which makes accurate evaluation of right ventricular function quite difficult. Therefore, our results may have certain flaws. Elevated NT-proBNP levels are evidence of clinical HF or increased wall stress, which might be caused by elevated ventricular filling pressure (33, 34). In this study, DCM patients with severe OSA had significantly greater NT-proBNP levels than patients with mild to moderate OSA and those without OSA. More importantly, NT-proBNP was independently correlated with the AHI. These findings provide a clue that OSA may aggravate the degree of HF in DCM patients.

OSA has been implicated as a contributor to the prevalence and severity of CVD. However, CPAP treatment did not reduce the risk of vascular outcomes for patients with OSA in recent trials (35–37). Therefore, it is essential to develop a novel strategy to counteract the pathophysiological mechanisms of the adverse consequences associated with OSA. One study reported that cardiac fibroblasts promote the hypertrophic response of cardiomyocytes through the transfer of exosomal miR-21 (38). MiR-200a is abundantly expressed in adipose tissue, inhibits TSC1 (a repressor of mTOR signaling) expression, promotes mTOR expression and leads to cardiomyocyte hypertrophy (39). Circulating exosomes derived from untreated OSA patients significantly increased endothelial cell senescence and endothelial dysfunction (24). These results suggested that OSA-derived exosomes and their cargo may be associated with cardiomyocyte injury. Thus, we studied the effects of exosomes derived from untreated OSA patients on cardiomyocyte fibrosis and hypertrophy and explored the possible mechanism.

In our study, we found that Col1A1, ANP and BNP expression levels were significantly increased with OSA-Exos treatment. These results demonstrate that exosomes may be a mediator to induce cardiomyocyte injury.

Autophagy is a highly conservative process that contributes to the elimination of superfluous materials and plays an important role in cardiac hypertrophy (40). Decreased autophagy disrupts energy and protein metabolism and induces severe cardiac hypertrophy (41), whereas the autophagy inducer rapamycin reduces aging-induced cardiac remodeling and hypertrophy (42). Therefore, we hypothesized that autophagy was also involved in OSA-Exos-induced cardiomyocyte injury. Our results confirmed that the level of autophagy decreased with OSA-Exos treatment as characterized by a decreased ratio of LC3B II/I and beclin1 expression.

mTOR, a serine/threonine kinase, promotes cell growth and autophagy *via* various pathways (43). mTOR is a critical downstream target of Akt, which plays an important role in extracellular matrix synthesis and cardiomyocyte hypertrophy in the progression of HF (44). In our experiments, p-Akt/Akt and p-mTOR/mTOR in our experiments, levels were increased by OSA-Exos treatment. These results illustrate that OSA-Exos also inhibited autophagy through the Akt/mTOR signaling pathway. In addition, rapamycin significantly decreased the elevated expression of Col1A1, ANP and BNP induced by OSA-Exos treatments and alleviated OSA-Exos-induced cardiomyocyte injury. Taken together, OSA-Exos promoted cardiomyocyte injury *via* Akt/mTOR signaling pathway-mediated autophagy.

There are several limitations in this study that merit discussion. First, this is a single-center retrospective study with a relatively small sample size; therefore, selection bias may exist in this study. Further prospective studies with a larger cohort of subjects are needed to support the present findings and investigate the exact effects of OSA on HF. Additionally, given the lack of data on CPAP treatment, whether the changes in cardiac structure and function will be reversed by CPAP treatment has not been confirmed; studies involving OSA-derived exosomes from pre- and post- treated patients are also needed to elucidate the effects and possible mechanism of OSA-Exos.

## Conclusions

Based on the clinical data, we showed that DCM patients with severe OSA had larger RVEDd and RAD and increased NT-proBNP levels than DCM patients without OSA. Moreover, NT-proBNP and diabetes mellitus were independently correlated with the AHI in multiple linear regression analysis. The AHI is significantly associated with cardiovascular remodeling and may be a potential variable to predict cardiac dysfunction and remodeling. In basic experiment, we found that exosomes derived from OSA patients induce the expression of HF markers in cardiomyocytes, and this process may be related to the decreased autophagy mediated by the Akt/mTOR signaling pathway. Identification of critical elements within exosomal cargo in OSA patients with accelerated HF may enable the development of novel therapeutic strategies for these patients.

## Data Availability Statement

The original contributions presented in the study are included in the article/Supplementary Material, further inquiries can be directed to the corresponding author/s.

## Ethics Statement

The studies involving human participants were reviewed and approved by the Institutional Ethics Committee of the Second Xiangya Hospital of Central South University. The patients/participants provided their written informed consent to participate in this study.

## Author Contributions

XZ, HG, and XL are the principal investigators, conducted statistical analysis, and drafted the article. LD and ST performed data management. SL, JP, and YL edited and revised the article. All authors read and approved the final article. All authors contributed to the article and approved the submitted version.

## Funding

This work was supported by the National Natural Science Foundation of China (No. 81470256), the Science and Technology Plan Project of the Changsha City (No. kq2001040), the Scientific Research Project of Hunan Science and Technology Department (No. 2018SK52510), and the Foundation Research Funds for the Central Universities of Central South University (2019zzts358).

## Conflict of Interest

The authors declare that the research was conducted in the absence of any commercial or financial relationships that could be construed as a potential conflict of interest.

## Publisher's Note

All claims expressed in this article are solely those of the authors and do not necessarily represent those of their affiliated organizations, or those of the publisher, the editors and the reviewers. Any product that may be evaluated in this article, or claim that may be made by its manufacturer, is not guaranteed or endorsed by the publisher.

## References

[1] Weintraub RG Semsarian C Macdonald P. Dilated cardiomyopathy. Lancet. (2017) 390:400–14. 10.1016/S0140-6736(16)31713-5 28190577

[2] Hershberger RE Hedges DJ Morales A. Dilated cardiomyopathy: the complexity of a diverse genetic architecture. Nat Rev Cardiol. (2013) 10:531–47. 10.1038/nrcardio.2013.105 23900355

[3] Reichart D Magnussen C Zeller T Blankenberg S. Dilated cardiomyopathy: from epidemiologic to genetic phenotypes: a translational review of current literature. J Intern Med. (2019) 286:362–72. 10.1111/joim.12944 31132311

[4] McKenna WJ Maron BJ Thiene G. Classification, epidemiology, and global burden of cardiomyopathies. Circ Res. (2017) 121:722–30. 10.1161/CIRCRESAHA.117.309711 28912179

[5] Gottlieb DJ Punjabi NM. Diagnosis and management of obstructive sleep apnea: a review. JAMA. (2020) 323:1389–400. 10.1001/jama.2020.3514 32286648

[6] Parati G Lombardi C Castagna F Mattaliano P Filardi PP Agostoni P. Heart failure and sleep disorders. Nat Rev Cardiol. (2016) 13:389–403. 10.1038/nrcardio.2016.71 27173772

[7] Khalyfa A Castro-Grattoni AL Gozal D. Cardiovascular morbidities of obstructive sleep apnea and the role of circulating extracellular vesicles. Ther Adv Respir Dis. (2019) 13:1753466619895229. 10.1177/1753466619895229 31852426 PMC6923690

[8] Banno K Shiomi T Sasanabe R Otake K Hasegawa R Maekawa M. Sleep-disordered breathing in patients with idiopathic cardiomyopathy. Circ J. (2004) 68:338–42. 10.1253/circj.68.338 15056831

[9] Chen RK Hong C Zhou YM Kuang AL Zhang YT Qing SM. [Severe obstructive sleep apnea-hypopnea syndrome with dilated cardiomyopathy leading to pulmonary hypertension: case report and literature review]. Zhonghua Jie He He Hu Xi Za Zhi. (2017) 40:46–51. 28100362 10.3760/cma.j.issn.1001-0939.2017.01.010

[10] Usui K Parker JD Newton GE Floras JS Ryan CM Bradley TD. Left ventricular structural adaptations to obstructive sleep apnea in dilated cardiomyopathy. Am J Respir Crit Care Med. (2006) 173:1170–5. 10.1164/rccm.200503-320OC 16514115

[11] Malone S Liu PP Holloway R Rutherford R Xie A Bradley TD. Obstructive sleep apnoea in patients with dilated cardiomyopathy: effects of continuous positive airway pressure. Lancet. (1991) 338:1480–4. 10.1016/0140-6736(91)92299-H 1683918

[12] Stewart S Ekman I Ekman T Oden A Rosengren A. Population impact of heart failure and the most common forms of cancer: a study of 1 162 309 hospital cases in Sweden (1988 to 2004). Circ Cardiovasc Qual Outcomes. (2010) 3:573–80. 10.1161/CIRCOUTCOMES.110.957571 20923990

[13] Desai AS Stevenson LW. Rehospitalization for heart failure: predict or prevent? Circulation. (2012) 126:501–6. 10.1161/CIRCULATIONAHA.112.125435 22825412

[14] Tual-Chalot S Gagnadoux F Trzepizur W Priou P Andriantsitohaina R Martinez MC. Circulating microparticles from obstructive sleep apnea syndrome patients induce endothelin-mediated angiogenesis. Biochim Biophys Acta. (2014) 1842:202–7. 10.1016/j.bbadis.2013.11.017 24275556

[15] Ayers L Stoewhas AC Ferry B Stradling J Kohler M. Elevated levels of endothelial cell-derived microparticles following short-term withdrawal of continuous positive airway pressure in patients with obstructive sleep apnea: data from a randomized controlled trial. Respiration. (2013) 85:478–85. 10.1159/000342877 23154449

[16] Jia S Zocco D Samuels ML Chou MF Chammas R Skog J. Emerging technologies in extracellular vesicle-based molecular diagnostics. Expert Rev Mol Diagn. (2014) 14:307–21. 10.1586/14737159.2014.893828 24575799

[17] Vlassov AV Magdaleno S Setterquist R Conrad R. Exosomes: current knowledge of their composition, biological functions, and diagnostic and therapeutic potentials. Biochim Biophys Acta. (2012) 1820:940–8. 10.1016/j.bbagen.2012.03.017 22503788

[18] Bagno L Hatzistergos KE Balkan W Hare JM. Mesenchymal stem cell-based therapy for cardiovascular disease: progress and challenges. Mol Ther. (2018) 26:1610–23. 10.1016/j.ymthe.2018.05.009 29807782 PMC6037203

[19] Ferguson SW Nguyen J. Exosomes as therapeutics: The implications of molecular composition and exosomal heterogeneity. J Control Release. (2016) 228:179–90. 10.1016/j.jconrel.2016.02.037 26941033

[20] Rayner KJ Hennessy EJ. Extracellular communication *via* microRNA: lipid particles have a new message. J Lipid Res. (2013) 54:1174–81. 10.1194/jlr.R034991 23505318 PMC3622315

[21] Christianson HC Svensson KJ van Kuppevelt TH Li JP Belting M. Cancer cell exosomes depend on cell-surface heparan sulfate proteoglycans for their internalization and functional activity. Proc Natl Acad Sci U S A. (2013) 110:17380–5. 10.1073/pnas.1304266110 24101524 PMC3808637

[22] Ailawadi S Wang X Gu H Fan GC. Pathologic function and therapeutic potential of exosomes in cardiovascular disease. Biochim Biophys Acta. (2015) 1852:1–11. 10.1016/j.bbadis.2014.10.008 25463630 PMC4268281

[23] Khalyfa A Zhang C Khalyfa AA Foster GE Beaudin AE Andrade J. Effect on intermittent hypoxia on plasma exosomal micro RNA signature and endothelial function in healthy adults. Sleep. (2016) 39:2077–90. 10.5665/sleep.6302 27634792 PMC5103796

[24] Khalyfa A Marin JM Qiao Z Rubio DS Kheirandish-Gozal L Gozal D. Plasma exosomes in OSA patients promote endothelial senescence: effect of long-term adherent continuous positive airway pressure. Sleep. (2020) 43(2). 10.1093/sleep/zsz217 31552414 PMC7901815

[25] Yancy CW Jessup M Bozkurt B Butler J Casey DE Drazner MH. ACCF/AHA guideline for the management of heart failure: executive summary: a report of the American College of Cardiology Foundation/American Heart Association Task Force on practice guidelines. Circulation. (2013) 128:1810–52. 10.1161/CIR.0b013e31829e8807 23741057

[26] Al-Bari MAA Xu P. Molecular regulation of autophagy machinery by mTOR-dependent and -independent pathways. Ann N Y Acad Sci. (2020) 1467:3–20. 10.1111/nyas.14305 31985829

[27] Heinzer R Vat S Marques-Vidal P Marti-Soler H Andries D Tobback N. Prevalence of sleep-disordered breathing in the general population: the HypnoLaus study. Lancet Respir Med. (2015) 3:310–8. 10.1016/S2213-2600(15)00043-0 25682233 PMC4404207

[28] Ding Q Qin L Wojeck B Inzucchi SE Ibrahim A Bravata DM. Polysomnographic phenotypes of obstructive sleep apnea and incident type 2 diabetes: results from the DREAM study. Ann Am Thorac Soc. (2021) 21:679. 10.1164/ajrccm-conference.2021.203.1_MeetingAbstracts.A4679 34185617 PMC8641817

[29] Bradley TD Floras JS. Sleep apnea and heart failure: Part I: obstructive sleep apnea. Circulation. (2003) 107:1671–8. 10.1161/01.CIR.0000061757.12581.15 12668504

[30] Shivalkar B Van de Heyning C Kerremans M Rinkevich D Verbraecken J De Backer W. Obstructive sleep apnea syndrome: more insights on structural and functional cardiac alterations, and the effects of treatment with continuous positive airway pressure. J Am Coll Cardiol. (2006) 47:1433–9. 10.1016/j.jacc.2005.11.054 16580533

[31] Brinker JA Weiss JL Lappe DL Rabson JL Summer WR Permutt S. Leftward septal displacement during right ventricular loading in man. Circulation. (1980) 61:626–33. 10.1161/01.CIR.61.3.626 7353253

[32] Tarasiuk A Scharf SM. Cardiovascular effects of periodic obstructive and central apneas in dogs. Am J Respir Crit Care Med. (1994) 150:83–9. 10.1164/ajrccm.150.1.8025778 8025778

[33] Ibrahim NE Gaggin HK Konstam MA Januzzi JL. Established and Emerging Roles of Biomarkers in Heart Failure Clinical Trials. Circ Heart Fail. (2016) 9:2528. 10.1161/CIRCHEARTFAILURE.115.002528 27582282

[34] Daubert MA Adams K Yow E Barnhart HX Douglas PS Rimmer S. NT-proBNP goal achievement is associated with significant reverse remodeling and improved clinical outcomes in HFrEF. JACC Heart Fail. (2019) 7:158–68. 10.1016/j.jchf.2018.10.014 30611722

[35] Yu J Zhou Z McEvoy RD Anderson CS Rodgers A Perkovic V. Association of positive airway pressure with cardiovascular events and death in adults with sleep apnea: a systematic review and meta-analysis. JAMA. (2017) 318:156–66. 10.1001/jama.2017.7967 28697252 PMC5541330

[36] da Silva Paulitsch F Zhang L. Continuous positive airway pressure for adults with obstructive sleep apnea and cardiovascular disease: a meta-analysis of randomized trials. Sleep Med. (2019) 54:28–34. 10.1016/j.sleep.2018.09.030 30529774

[37] Drager LF McEvoy RD Barbe F Lorenzi-Filho G Redline S Initiative I. Sleep apnea and cardiovascular disease: lessons from recent trials and need for team science. Circulation. (2017) 136:1840–50. 10.1161/CIRCULATIONAHA.117.029400 29109195 PMC5689452

[38] Bang C Batkai S Dangwal S Gupta SK Foinquinos A Holzmann A. Cardiac fibroblast-derived microRNA passenger strand-enriched exosomes mediate cardiomyocyte hypertrophy. J Clin Invest. (2014) 124:2136–46. 10.1172/JCI70577 24743145 PMC4001534

[39] Fang X Stroud MJ Ouyang K Fang L Zhang J Dalton ND. Adipocyte-specific loss of PPARgamma attenuates cardiac hypertrophy. JCI Insight. (2016) 1:e89908. 10.1172/jci.insight.89908 27734035 PMC5053146

[40] Li L Xu J He L Peng L Zhong Q Chen L. The role of autophagy in cardiac hypertrophy. Acta Biochim Biophys Sin (Shanghai). (2016) 48:491–500. 10.1093/abbs/gmw025 27084518 PMC4913516

[41] Zaglia T Milan G Ruhs A Franzoso M Bertaggia E Pianca N. Atrogin-1 deficiency promotes cardiomyopathy and premature death *via* impaired autophagy. J Clin Invest. (2014) 124:2410–24. 10.1172/JCI66339 24789905 PMC4038560

[42] Hua Y Zhang Y Ceylan-Isik AF Wold LE Nunn JM Ren J. Chronic Akt activation accentuates aging-induced cardiac hypertrophy and myocardial contractile dysfunction: role of autophagy. Basic Res Cardiol. (2011) 106:1173–91. 10.1007/s00395-011-0222-8 21901288

[43] Saxton RA Sabatini DM. mTOR signaling in growth, metabolism, and disease. Cell. (2017) 168:960–76. 10.1016/j.cell.2017.02.004 28283069 PMC5394987

[44] Sciarretta S Forte M Frati G Sadoshima J. New insights into the role of mtor signaling in the cardiovascular system. Circ Res. (2018) 122:489–505. 10.1161/CIRCRESAHA.117.311147 29420210 PMC6398933

