# Supplementary material for: Obstructive Sleep Apnea Impacts Cardiac Function in Dilated Cardiomyopathy Patients Through Circulating Exosomes
Source: Front Cardiovasc Med. 2022 Mar 7;9:699764. doi: 10.3389/fcvm.2022.699764 (PMC8936144; doi:10.3389/fcvm.2022.699764)
Supplement: Supplementary file 1 [file Data_Sheet_1.doc]

Supplementary Table1. The baseline characteristics of the enrolled patients

| Characteristics | Control (n=10) | OSA patients (n=10) | *p* value |
| --- | --- | --- | --- |
| Age (year) | 34.40±7.48 | 43.40±8.57 | 0.022 |
| Sex (Male, %) | 8 (80%) | 9 (90%) | 0.531 |
| BMI (kg/m^2^) | 21.98±1.84 | 26.50±2.89 | 0.001 |
| AHI (events/h) | 3.20 (2.18, 3.85) | 38.5 (30.3, 52.3) | 0.000 |
| SBP（mmHg） | 120.20±13.61 | 124.10±12.15 | 0.508 |
| DBP（mmHg） | 78±8.31 | 83.80±4.85 | 0.073 |
| ALT (μ/L) | 18.24±4.90 | 20.74±8.17 | 0.418 |
| AST (μ/L) | 21.26±5.21 | 24.07±7.11 | 0.327 |
| Cholesterol (mmol/L) | 3.80 (3.50, 4.10) | 4.23 (3.61, 4.33) | 0.082 |
| Triglyceride (mmol/L) | 1.50 (1.12, 1.67) | 1.41 (1.23, 1.74) | 0.796 |
| HDL-C (mmol/L) | 0.89 (0.73, 0.98) | 1.04 (0.79, 1.14) | 0.063 |
| LDL-C (mmol/L) | 2.53 (1.85, 3.07) | 2.63 (2.16, 2.90) | 0.853 |
| Glucose (mmol/L) | 5.20 (5.08, 5.53) | 5.25 (4.78, 5.78) | 1.000 |

Data are expressed as means ±standard deviation for normally distributed data, median (interquartile range) for nonnormally distributed data, or number (%) for categorical variables.

Abbreviations are as shown in Table1.

Supplementary Table2. Correlation analysis and multivariate stepwise regression analysis of the factors on the presence of OSA

| Factors | Correlation analysis | | Multivariate stepwise regression analysis | |
| --- | --- | --- | --- | --- |
|  | r | *p* | Standardized β (95% CI) | *p* |
| Age | 0.426 | 0.061 | / | / |
| Sex | -0.140 | 0.556 | / | / |
| BMI | 0.703 | 0.001 | 0.068(-0.036,0.057) | 0.636 |
| AHI | 0.867 | 0.000 | 0.814(0.012,0.027) | 0.000 |
| SBP | 0.217 | 0.358 | / | / |
| DBP | 0.565 | 0.009 | 0.157(-0.004,0.057) | 0.141 |
| ALT | 0.113 | 0.636 | / | / |
| AST | 0.295 | 0.207 | / | / |
| Cholesterol | 0.400 | 0.081 | / | / |
| Triglyceride | 0.061 | 0.799 | / | / |
| HDL-C | 0.434 | 0.056 | / | / |
| LDL-C | 0.052 | 0.827 | / | / |
| Glucose | 0.000 | 1.000 | / | / |

Abbreviations are as shown in Table1.
