# Supplementary material for: Obstructive Sleep Apnea Impacts Cardiac Function in Dilated Cardiomyopathy Patients Through Circulating Exosomes
Source: Front Cardiovasc Med. 2022 Mar 7;9:699764. doi: 10.3389/fcvm.2022.699764 (PMC8936144; doi:10.3389/fcvm.2022.699764)
Supplement: Supplementary file 2 [file Data_Sheet_2.docx]

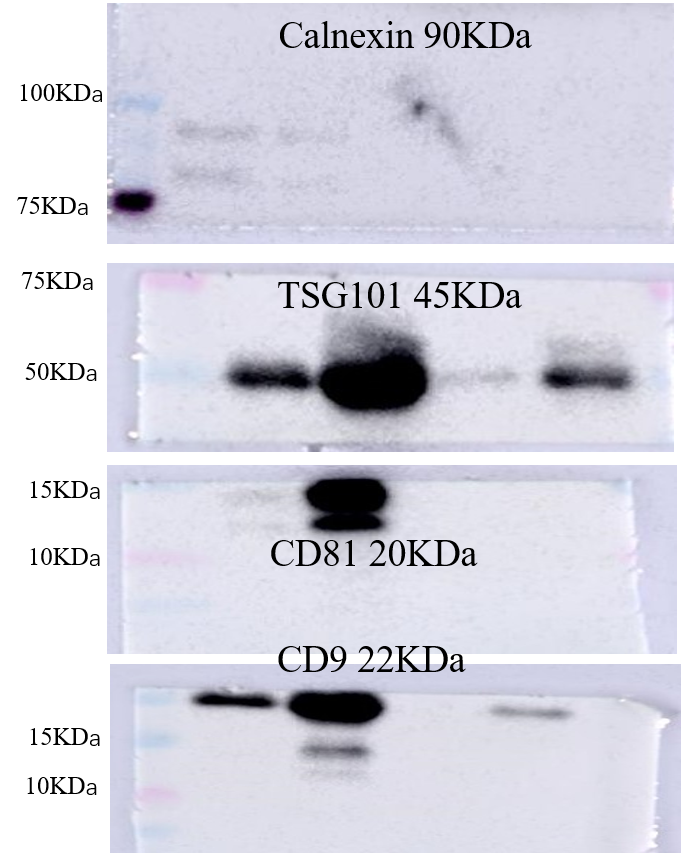


Figure1. shows the exosomal markers after cutting membrane at molecular weight 100~75 kDa, 75~50 kDa, and 15~10 kDa for Calnexin (90 kDa), TSG101 (45 kDa), CD81 (20kDa), and CD9 (22 kDa).


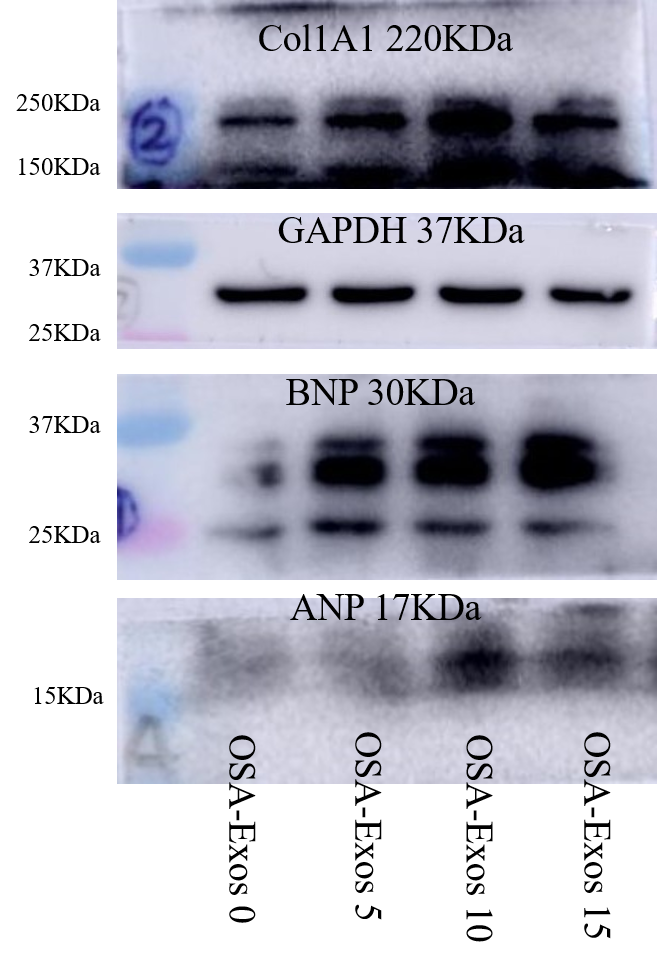


Figure2. shows the cardiomyocyte fibrosis and hypertrophy markers after cutting membrane at molecular weight 250~150 kDa, 37~25 kDa, 25KDa and 15 kDa for Col1A1 (220 kDa), GAPDH (37 kDa), BNP (30kDa), and ANP (17 kDa).


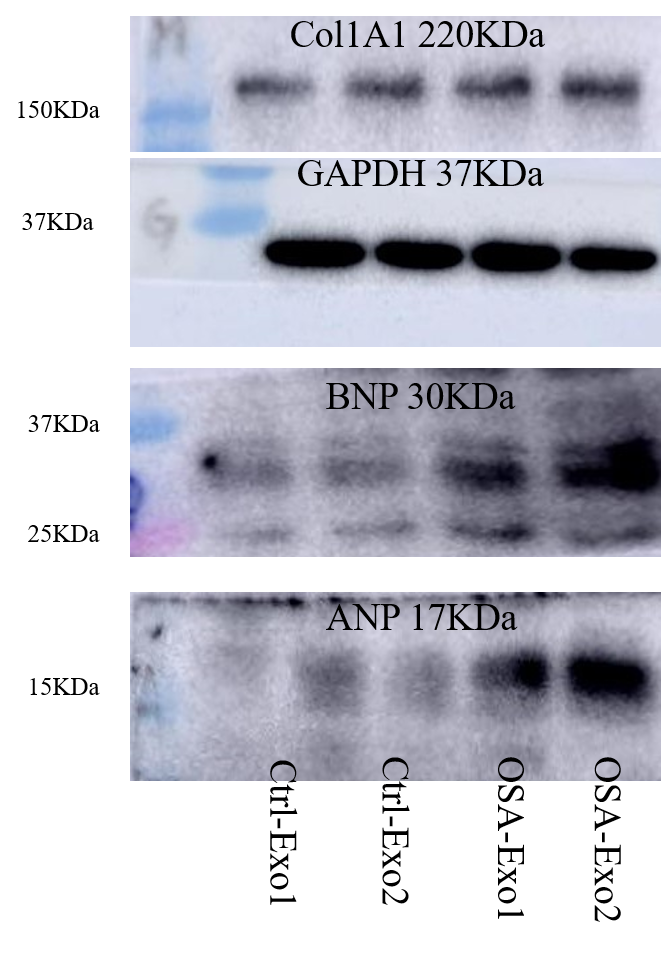


Figure3. shows the cardiomyocyte fibrosis and hypertrophy markers after cutting membrane at molecular weight 250~150 kDa, 37~25 kDa, 25KDa and 15 kDa for Col1A1 (220 kDa), GAPDH (37 kDa), BNP (30kDa), and ANP (17 kDa).


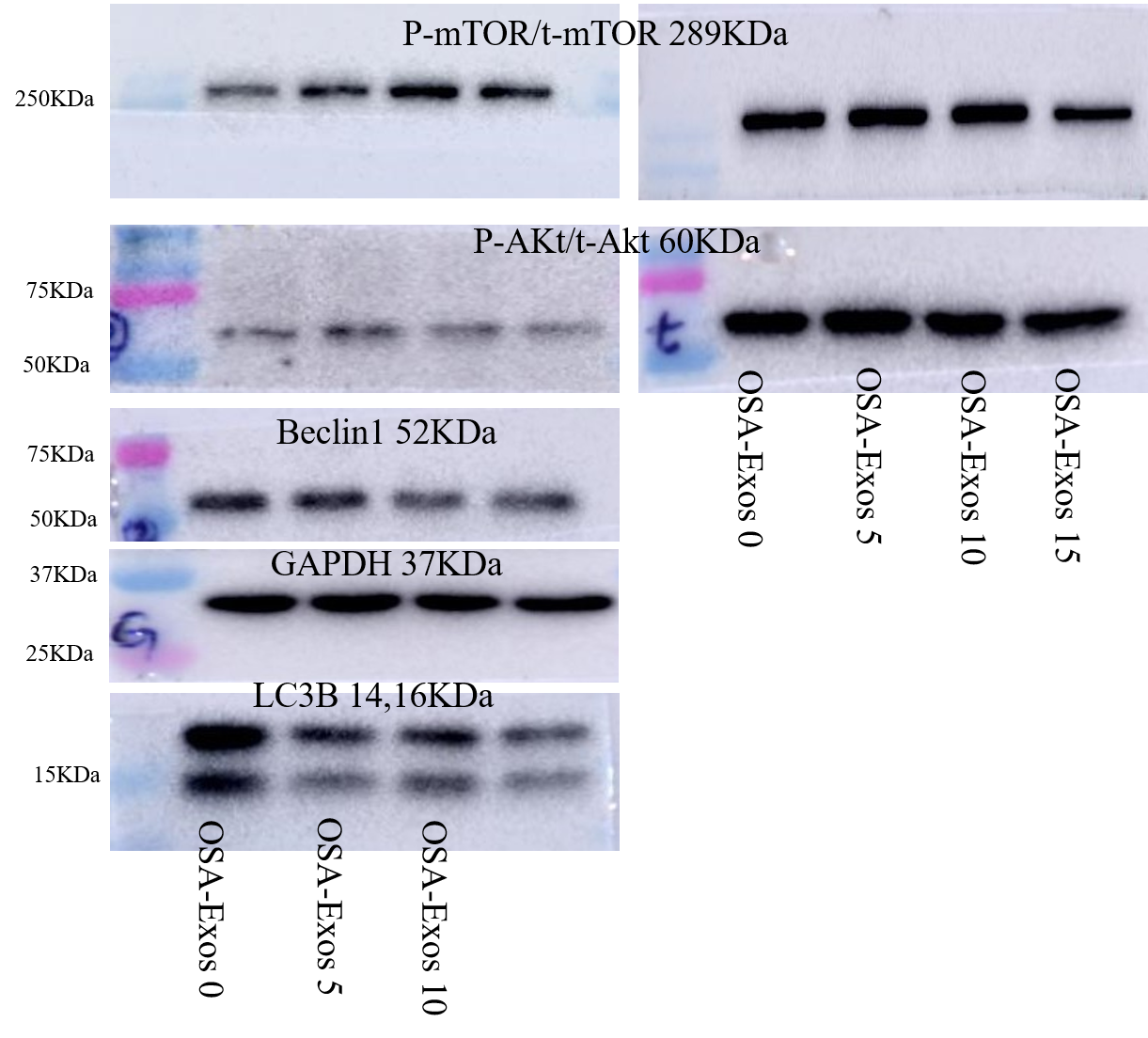


Figure4. shows the Akt/mTOR signaling regulated autophagy proteins expression after cutting membrane at molecular weight 250kDa, 75~50 kDa, 37~25KDa and 15 kDa for p-Mtor/Motr (289 kDa), p-Akt/Akt (60 kDa), beclin1(52KDa), GAPDH (37KDa) and LC3B (14,16 kDa).


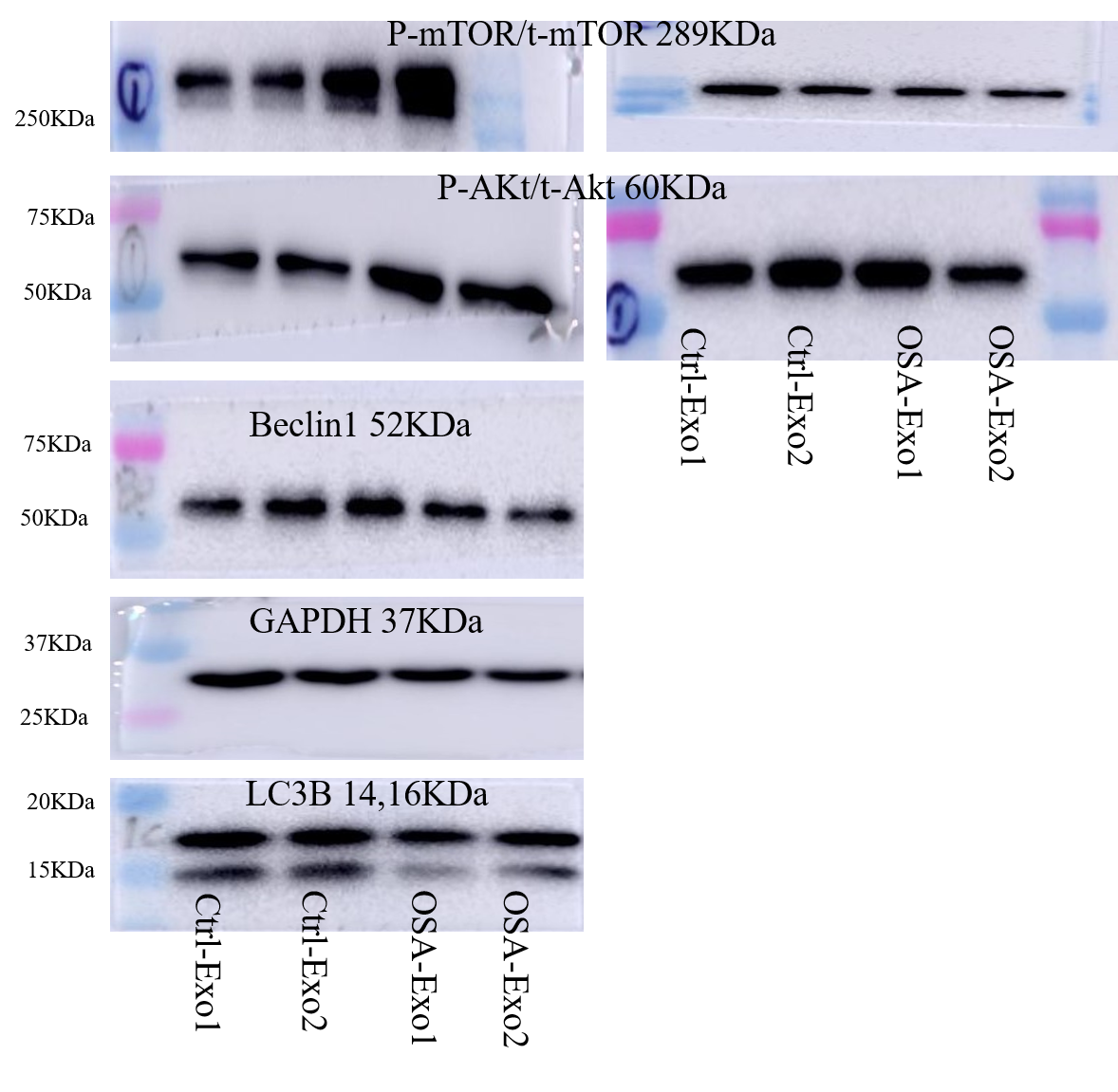


Figure5. shows the Akt/mTOR signaling regulated autophagy proteins expression after cutting membrane at molecular weight 250kDa, 75~50 kDa, 37~25KDa and 15 kDa for p-mTOR/mTOR (289 kDa), p-Akt/Akt (60 kDa), beclin1(52KDa), GAPDH (37KDa) and LC3B (14,16 kDa).


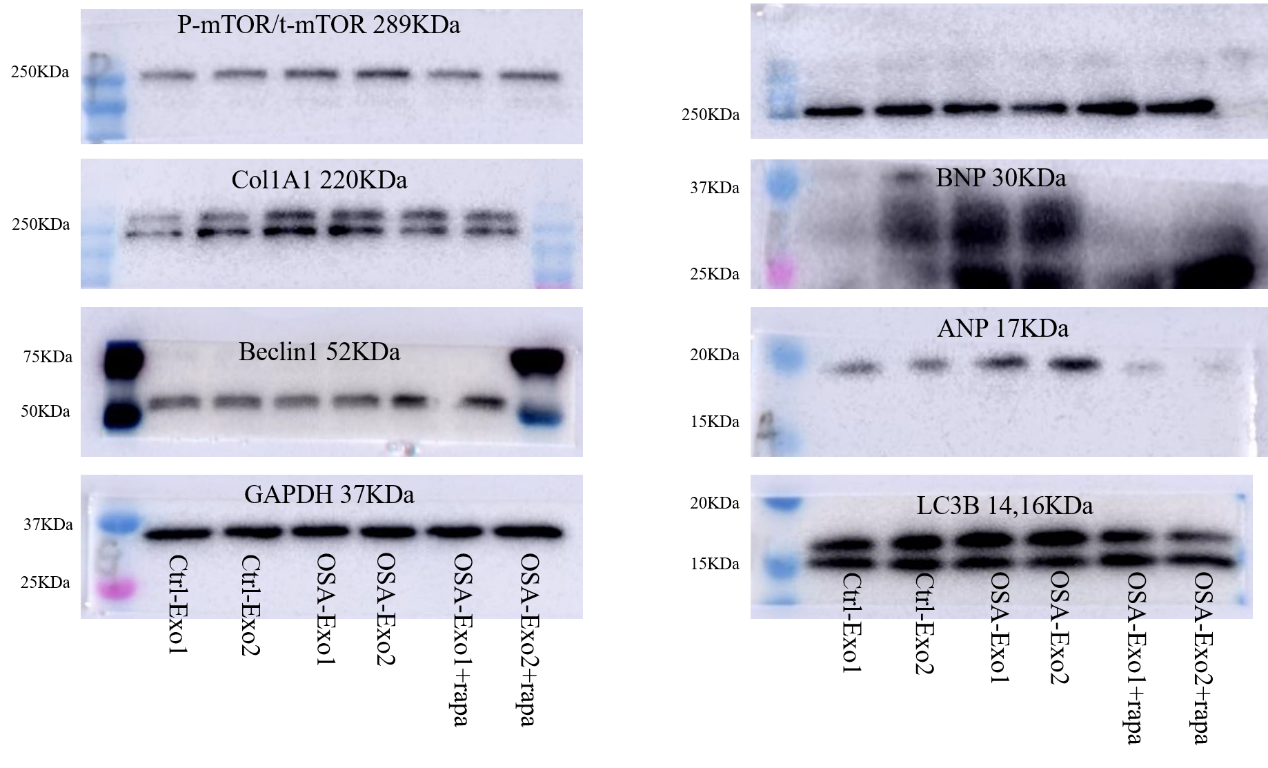


Figure6. shows the Akt/mTOR signaling regulated autophagy proteins expression after cutting membrane at molecular weight 250kDa, 75~50 kDa, 37~25KDa and 20~15 kDa for p-mTOR/mTOR (289 kDa), Col1A1 (220KDa), beclin1(52KDa), GAPDH (37KDa), ANP (17KDa), BNP (30KDa) and LC3B (14,16 kDa).
